# Altered Fecal Microbiota Signatures in Patients With Anxiety and Depression in the Gastrointestinal Cancer Screening: A Case-Control Study

**DOI:** 10.3389/fpsyt.2021.757139

**Published:** 2021-11-08

**Authors:** Juan Zhu, Minjuan Li, Dantong Shao, Shanrui Ma, Wenqiang Wei

**Affiliations:** National Central Cancer Registry, National Cancer Center/National Clinical Research Center for Cancer/Cancer Hospital, Chinese Academy of Medical Sciences and Peking Union Medical College, Beijing, China

**Keywords:** anxiety, depression, gut microbiota, 16S rRNA gene sequencing, microbiota-gut-brain axis, gastrointestinal cancer, endoscopic screening

## Abstract

**Background:** Increasing attention has been devoted to cancer screening and microbiota in recent decades, but currently there is less focus on microbiota characterization among screeners and its relationship to anxiety and depression.

**Methods:** We characterized the microbial communities of fecal samples collected through the FOBT card from anxiety and depression screeners and paired controls in Henan, China (1:2, *N* = 69). DNA was extracted using the MOBIO PowerSoil kit. The V4 region of the 16S rRNA gene was sequenced using MiniSeq and processed using QIIME1. LEfSe was used to identify differentially abundant microbes, the Wilcoxon rank-sum test was used to test alpha diversity differences, and permutational multivariate analysis of variance was used to test for differences in beta diversity.

**Results:** Similar fecal microbiota signatures in composition were found among screeners. The intestinal microbial environments by phylum were all composed primarily of *Firmicutes, Bacteroidetes*, and *Proteobacteria*, and the corresponding top genera were *Faecalibacterium, Roseburia*, and *Prevotella*. Compared with controls, the ranking of the top five genera in the anxiety and depression group changed, and the dominant genus was *Prevotella* in the anxiety and depression group and *Faecalibacterium* in the control group. There was a lower relative abundance of *Gemmiger* (1.4 vs. 2.3%, *P* = 0.025), *Ruminococcus* (0.6 vs. 0.8%, *P* = 0.037), and *Veillonella* (0.6 vs. 1.3%, *P* = 0.020). This may be linked to the lower alpha diversity in participants with anxiety and depression (Observed OTUs: 122.35 vs. 143.24; Chao1: 127.35 vs. 149.98), although no significant differences were observed. Distinct clustering in microbial composition between the two groups was detected for the Jaccard distance (*P* = 0.011).

**Conclusions:** Our study showed differing microbial characterization among participants with anxiety and depression in the endoscopic screening of upper gastrointestinal cancer. *Gemmiger, Ruminococcus*, and *Veillonella* were informative and have potential clinical implications, which need to be confirmed by large-scale, prospective cohort studies and biological mechanism research.

## Introduction

In excess of 100 trillion microorganisms colonize the human intestinal plot, which assumes a vital part in human wellbeing and illness conditions (1). The normal intestinal microbiota act as significant functions in host metabolism, xenobiotics, integrity maintenance of the intestinal mucosal barrier, immunomodulation, and assurance against microorganisms (2, 3). The microbiota-gut-brain axis, a research hotspot, refers to the bidirectional communication between the microorganisms residing in the gut and our brain function, behavior, and emotion (4, 5). Adequate evidence highlighted the multifaceted role of the intestinal microbiota in carcinogenesis (e.g., gastrointestinal cancer) and psychological distress (e.g., anxiety and depression disorders) (6–9). Previous research showed that anxiety and depression patients were characterized by a higher abundance of proinflammatory species (*Enterobacteriaceae* and *Desulfovibrio*), lower microbiota diversity, and a lower abundance of short-chain fatty acid-producing species (*Faecalibacterium*) (10).

Current national cancer screening recommendations referenced the potential harm of mental health owing to cancer screening (11). As people increase their emphasis on health problems, studies on cancer screening and psychology represent a growing field. Invasive endoscopic screening for gastrointestinal cancer is often accompanied by negative psychosocial consequences to participants (anxiety and depression symptoms) (12, 13). Considering the psychological distress in cancer screening and the microbiota-gut-brain axis, the relationship of microbiota and anxiety and depression among screeners becomes interesting. Discovering the microbial characteristics of screeners and microbiota diversity and characteristic genera affecting anxiety and depression would be valuable to optimize the strategy of cancer screening and reduce the negative psychological effects (anxiety and depression) caused by cancer screening. However, so far reliable evidence on microbiota characterization among screeners is limited and insufficient. No known research has investigated the relationship between intestinal microbiota and anxiety and depression among screeners. Therefore, the study aimed to explore the microbial characterization of participants in endoscopic screening and to identify psychological distress-associated gut microbiota.

## Materials and Methods

### Study Participants and Sample Collection

Based on the endoscopic screening of the National Cohort of Esophageal Cancer (NCEC) project in China, we retrospectively recruited permanent residents aged 40–69 years in August 2019. Fecal samples were collected before endoscopic screening of upper gastrointestinal cancer in Linzhou Cancer Hospital, Henan Province. Fecal sample collection process: (1) The fecal collection kit, including a fecal collection box and fecal occult blood test card (FOBT) for smearing feces, was prepared in advance. (2) The kit was distributed to the participants, and the sampling box was directly placed in the squatting stool by themselves. The fresh fecal collection was completed before endoscopy. (3) After defecation, the FOBT card was opened, the stool collection stick was used to pick up a small number of feces and smear them on the two panes of the FOBT card, and then the card was closed and the FOBT card was placed in the sealed bags. (4) The sealed bags were stored in the −80°C refrigerator in the biobank of Linzhou Cancer Hospital and shipped to the laboratory with dry ice.

Only people with both anxiety and depression symptoms were regarded as anxiety and depression screeners. Those who had neither anxiety nor depression symptoms were regarded as the control group (screeners without anxiety and depression). The control group was matched by age and sex (1:2). A total of 69 participants were included, with 23 anxiety and depression screeners and 46 paired screeners in the control group.

All participants signed written informed consent. This study was approved by the Institutional Review Board of the Cancer Hospital of the Chinese Academy of Medical Sciences (No. 16-171/1250). Participants' sociodemographic information was gathered by trained staff *via* a uniform questionnaire.

### Laboratory Handling and Bioinformatics (DNA Extraction, Amplification, and Sequencing)

Total bacterial deoxyribonucleic acid (DNA) was extracted from the fecal samples using the MOBIO PowerSoil® DNA Isolation Kit protocol. Barcoded amplicons were generated covering the V4 region of the 16S ribosomal RNA (16S rRNA) gene using the 515F (5′-GTGYCAGCMGCCGCGGTAA-3′) and 806R (5′-GGACTACNVGGGTWTCTAAT-3′) primers (14, 15). Polymerase chain reaction (PCR) mixtures contained 1 μL of forward and reverse primer, 1 μL of template DNA, 4 μL of deoxyribonucleoside-triphosphates (dNTPs), 5 μL of 10× EasyPfu Buffer, 1 μL of EasyPfu DNA Polymerase, and 1 μL of double distilled water into a 50 μL total reaction volume. The PCR amplicons were quantified using the Qubit dsDNA HS Assay Kit (Thermo Fisher/Invitrogen Cat. no. Q32854, Waltham, USA) following the manufacturer's instructions. All sequencing was acted in a solitary MiniSeq run and exported in the FASTQ format. Illumina MiniSeq Reporter was carried out to remove adapter and primer sequences.

All specimens collected were successfully amplified and sequenced. Sequencing data were performed with the Quantitative Insights into Microbial Ecology (QIIME2) platform (16). The raw sequences were processed to remove low-quality reads, under strict quality control and feature table construction using the Divisive Amplicon Denoising Algorithm 2 (DADA2) algorithm (17). A similarity threshold of 97% was matched. The taxonomic assignment of the sequence variants was assigned using the Greengenes 13_8 (18). The Shannon index rarefaction curve was represented in Supplementary Figure 1. A total of 23 positive anxiety and depression screeners with a mean of 51,272 reads and 46 non-anxiety and depression screeners with a mean of 60,697 reads were included in the analysis. Then we generated alpha diversity metrics and beta diversity metrics using QIIME.

### Measurement of Anxiety and Depression

The anxiety symptoms were evaluated by the seven-item Generalized Anxiety Disorder (GAD-7), a widely used and acknowledged measurement tool worldwide. Good psychometrics of GAD-7 has been proved in primary medical care (19). The reliability of internal consistency of GAD-7 in the study was strong (Cronbach's alpha = 0.888). GAD-7 was used to identify anxiety symptoms of individuals in the past 2 weeks, with seven items and four responses (0 = *never*; 1 = *sometimes*; 2 ≥ *half of day*; 3 = *almost every day*). The anxiety score was calculated by adding the answers for each of the items and ranges from 0 to 21. The higher the score, the worse anxious symptoms. A result of five was regarded as the threshold for positive anxiety symptoms (20).

The nine-item Patient Health Questionnaire (PHQ-9), one of the most well-known self-reported tools for assessing depression symptoms (21), has shown good performance for evaluating depressive disorder (22, 23). Cronbach's alpha coefficients of PHA-9 in our study were 0.896. PHQ-9 was used to identify depressive symptoms of individuals in the past 2 weeks, with nine items and four responses (similar to GAD-7). The PHQ-9 score was the sum of each item. The higher the score, the worse the depression symptoms. People with a PHQ-9 score of 5 or higher were considered positive for depression symptoms (23).

### Statistical Analysis

Chi-square tests and *T*-tests were used to compare basic characteristics between anxiety and depression screeners and the controls. Evenness index, Shannon index, Observed OTUs, and Chao1 index were used to reflect the alpha diversity. Differences in alpha diversity were analyzed between the anxiety and depression group and paired control group by Wilcoxon rank-sum test. Permutational Multivariate Analysis of Variance (PERMANOVA, R-vegan function adonis) was used to explore whether the flora composition differed by anxiety and depression status (beta diversity). Principal coordinate analysis (PCoA) was used to visualize clustering and find discrepancy among the independent β diversity matrices, based on Bary-Curtis dissimilarity, Jaccard distance, and weighted and unweighted unifrac distances. High relative abundance (≥0.01) genera were compared between the two groups by the Wilcoxon rank-sum test.

Linear discriminant analysis effect size (LEfSe) was used to identify microbes associated with anxiety and depression symptoms (24). Using Wilcoxon rank-sum test, LEfSe detects microbiota with significant differences between the two groups. Microbiota, with linear discriminant analysis scores (LDA) ≥ 2.032, were identified as potential characteristic flora associated with anxiety and depression symptoms. *P* < 0.05 were considered statistically significant. All analyses were conducted using the software program R Studio (Version 1.1.456).

## Results

### Baseline Characteristics of Participants

The average age of screeners in the anxiety and depression group and control group was 55.30 (SD = 8.20) and 55.63 (SD = 7.78), respectively. No statistically significant differences were observed for the baseline characteristics, including BMI, marital status, highest education level, household income, smoking status, alcohol drinking, hot food, and life satisfaction between the anxiety and depression group and the control group (see Table 1).

**Table 1:** Baseline characteristics of the anxiety and depression group and the control group.

**Characteristics**	**Total**		**Anxiety and depression group**		**Control group**		***P***
	**Frequency**	**%**	**Frequency**	**%**	**Frequency**	**%**	
*N*	69		23		46		
Age, year	55.52 ± 7.86		55.30 ± 8.20		55.63 ± 7.78		0.872
BMI, kg/m^2^	25.15 ± 3.40		24.43 ± 3.21		25.51 ± 3.47		0.213
Male	34	49.3	13	56.5	21	45.7	0.395
Married	66	95.7	21	91.3	45	97.8	0.210
**Highest education level**							0.114
Primary school or below	29	42	10	43.5	9	19.6	
Junior or senior high school	38	55.1	11	47.8	27	58.7	
Undergraduate or over	2	2.9	2	8.7	0	0	
**Household income (10,000 RMB/year)**							0.886
<3.0	12	17.4	4	17.4	8	17.4	
3.0–7.0	47	68.1	15	65.2	32	69.6	
7.0–11.0	10	14.5	4	17.4	6	13	
**Smoking status**							0.175
Do not smoke now	58	84.1	19	82.6	39	84.8	
Only occasionally	4	5.8	0	0	4	8.7	
Most days or almost every day	7	10.1	4	17.4	3	6.5	
**Alcohol consumption**							0.102
Never or almost never	61	88.4	21	91.3	40	87	
Only occasionally	5	7.2	0	0	5	10.9	
Most days or almost every day	3	4.3	2	8.7	1	2.2	
**Hot food (high temperature)**							0.549
Often	2	2.9	0	0	2	4.3	
Seldom	67	97.1	23	100	44	95.7	
**Life satisfaction**							0.084
Very satisfied	8	11.6	0	0	8	17.4	
Satisfied	58	84.1	22	95.7	36	78.3	
Just so so	3	4.3	1	4.3	2	4.3	

### Microbial Characterization of Participants in Endoscopic Screening

Similar fecal microbiota signatures in composition were found among screeners. The relative abundance of *Firmicutes* (relative abundance: 71.2%), *Bacteroidetes* (14.6%), *Proteobacteria* (5.8%), *Actinobacteria* (2.8%), and *Unknown* (1.8%) were the top five by phylum. The top five genera in specimens of participants of gastrointestinal cancer screening included *Faecalibacterium* (11.3%), *Roseburia* (10.4%), *Prevotella* (10.3%), *Blautia* (10.0%), and *Escherichia* (3.0%). As for alpha diversity, the Evenness index, Observed OTUs, Shannon index, and Chao1 index were 0.67, 135.77, 4.69, and 142.43. The results were displayed in Supplementary Table 1.

### Microbiota Characterization and Diversity of Screeners, by Anxiety and Depression

#### Alpha Diversity

**Figure 1:**
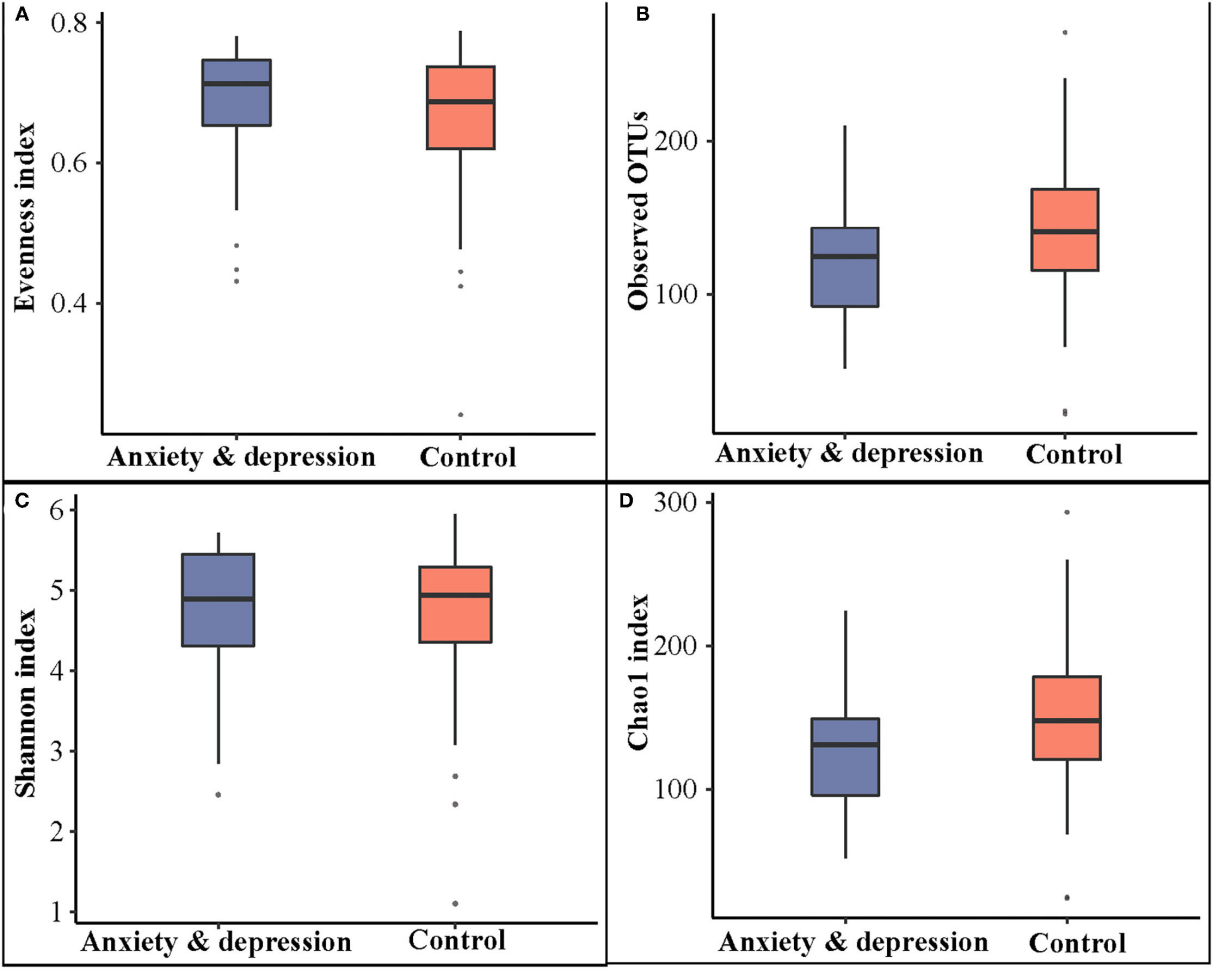
Microbial comparison between anxiety and depression group and control group for alpha diversity. **(A)** Evenness index, **(B)** observed OTUs, **(C)** Shannon index, and **(D)** Chao1 index.

As shown in Figure 1, the alpha diversity in the anxiety and depression group was decreased compared with the control group, although no significant differences were observed (Observed OTUs: 122.35 vs. 143.24; Shannon index: 4.66 vs. 4.70; Chao1 index: 127.35 vs. 149.98).

#### Beta Diversity

**Figure 2:**
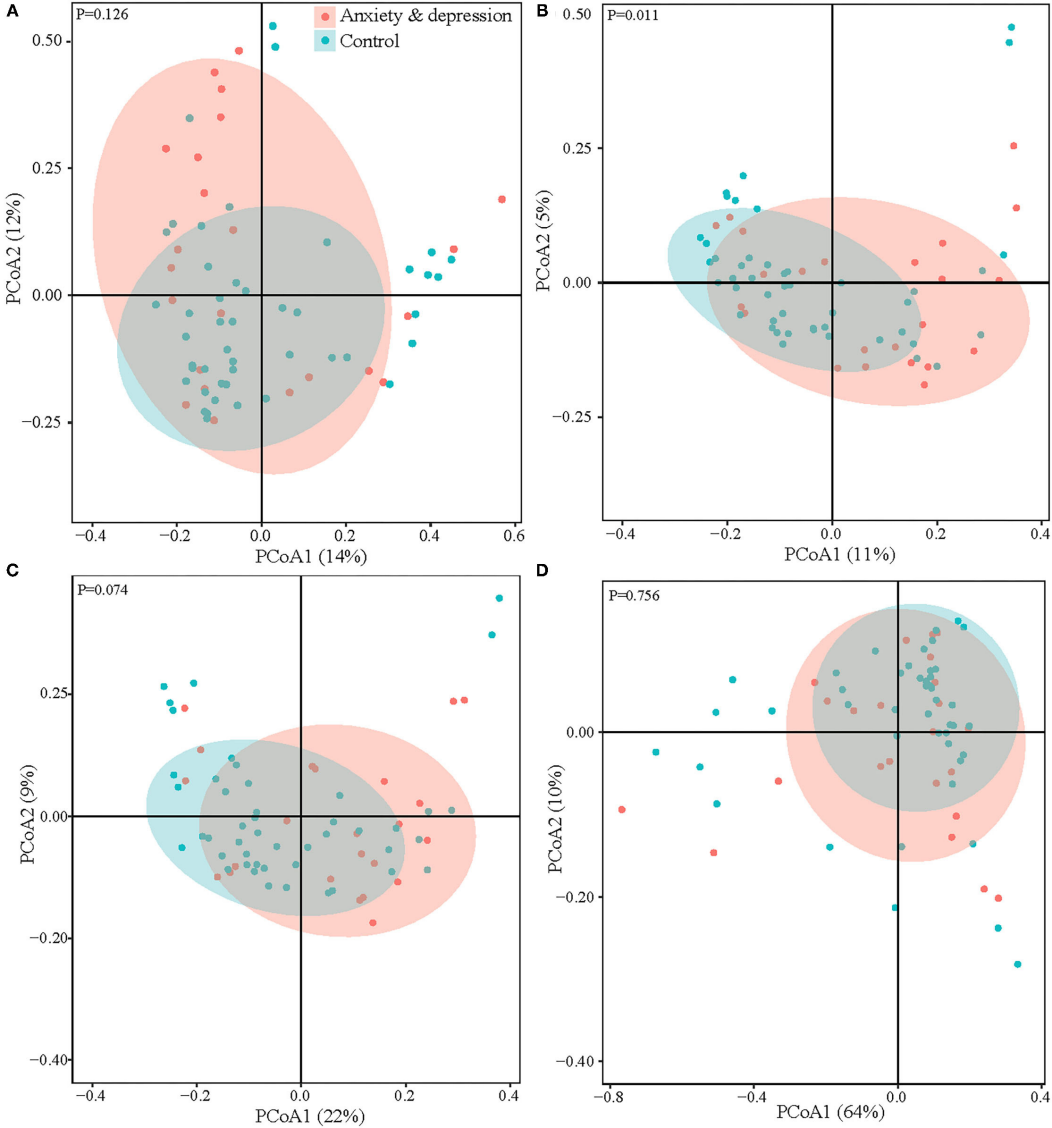
Microbial comparison between anxiety and depression group and control group for beta diversity. **(A)** Bray-curtis dissimilarity, **(B)** Jaccard distance, **(C)** unweighted UniFrac, and **(D)** weighted UniFrac.

When considering microbial community composition (i.e., beta diversity), significant clustering was found for the Jaccard distance (*P* = 0.011) between the anxiety and depression group and the control group but not for the Bray-Curtis dissimilarity, Weighted and Unweighted Unifrac distance (Figure 2).

#### Microbial Composition

**Figure 3:**
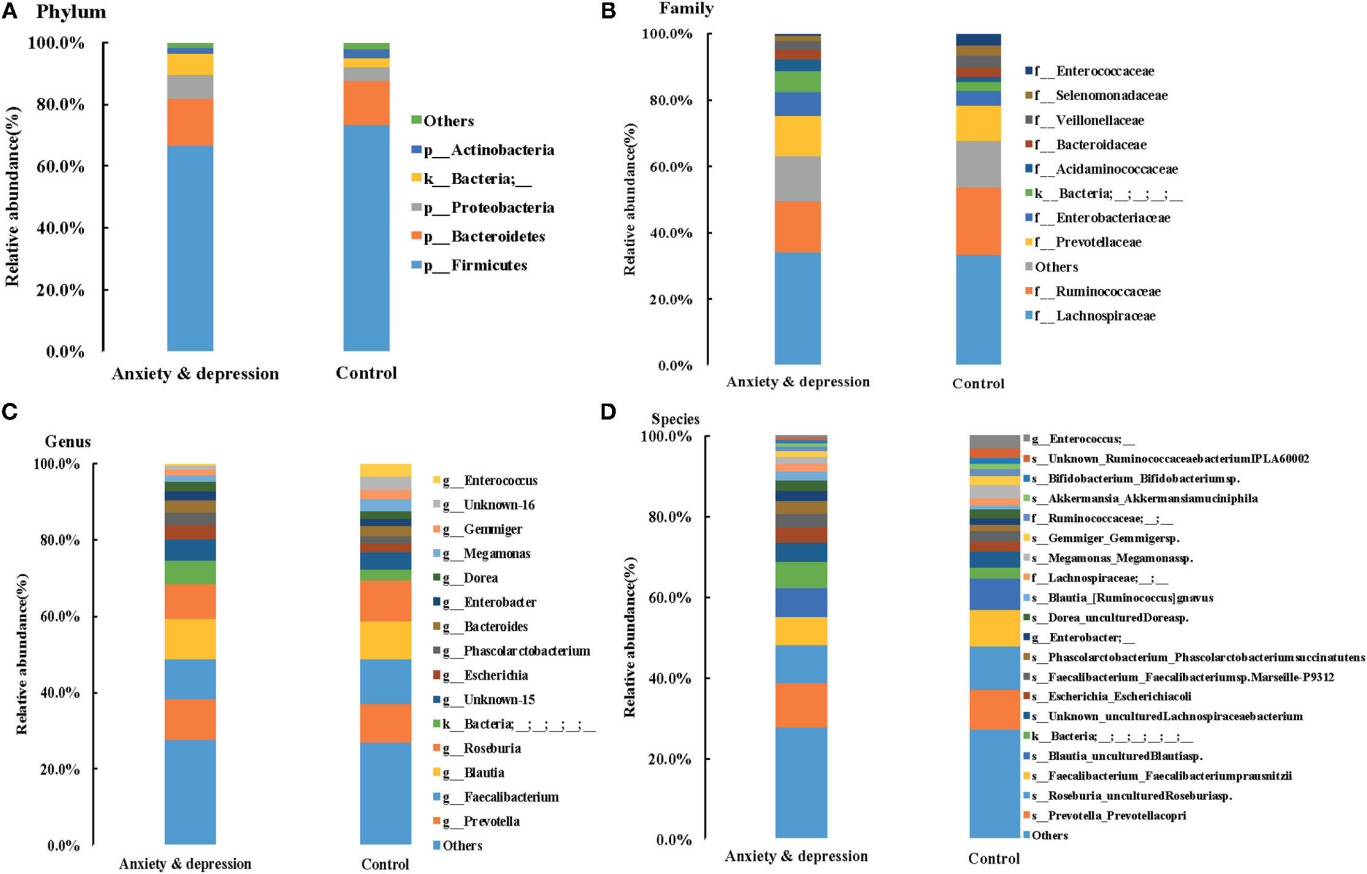
Microbial relative abundances for anxiety and depression group and control group. **(A)** Phylum, **(B)** Family, **(C)** Genus, and **(D)** Species.

Microbial relative abundances at the phylum, family, genus, and species levels for anxiety and depression group and control group were shown in Figure 3. Similar fecal microbiota signatures in composition were found between the two groups. At the phylum level, the intestinal microbial environments of the two groups were all comprised primarily of *Firmicutes* (66.6 vs. 73.4%, *P* = 0.240), *Bacteroidetes* (15.5 vs. 14.1%, *P* = 0.620), *Proteobacteria* (7.8 vs. 4.7%, *P* = 0.100), and *Actinobacteria* (2.2 vs.3.1%, *P* = 0.541). The *Bacteroidetes* and *Proteobacteria* in feces of patients with anxiety and depression increased, while the *Firmicutes* and *Actinobacteria* decreased, although there was no significant difference.

At the genus level, compared with controls, the *prevotella* and *Blautia* in feces of anxious and depressed patients showed an increasing trend, while the *Faecalibacterium* and *Roseburia* showed a downward trend, although the difference is not statistically significant. The relative abundance of *prevotella* (10.9 vs. 10.0%, *P* = 0.600), *Faecalibacterium* (10.5 vs. 11.8%, *P* = 0.470), *Blautia* (10.2 vs. 9.8%, *P* = 0.720), *Roseburia* (9.2 vs. 10.9%, *P* = 0.600), and *Escherichia* (3.9 vs. 2.6, *P* = 0.679) were the top five in both groups, but the ranking of the top five genera changed for subjects with anxiety and depression symptoms. The top one genera were *Prevotella* in the anxiety and depression group and *Faecalibacterium* in the control group (Figure 3 and Supplementary Table 1). Microbial relative abundances of each sample for the anxiety and depression group and control group were shown in Supplementary Figure 2.

#### Microbial Diversity and Characteristic Genus

**Figure 4:**
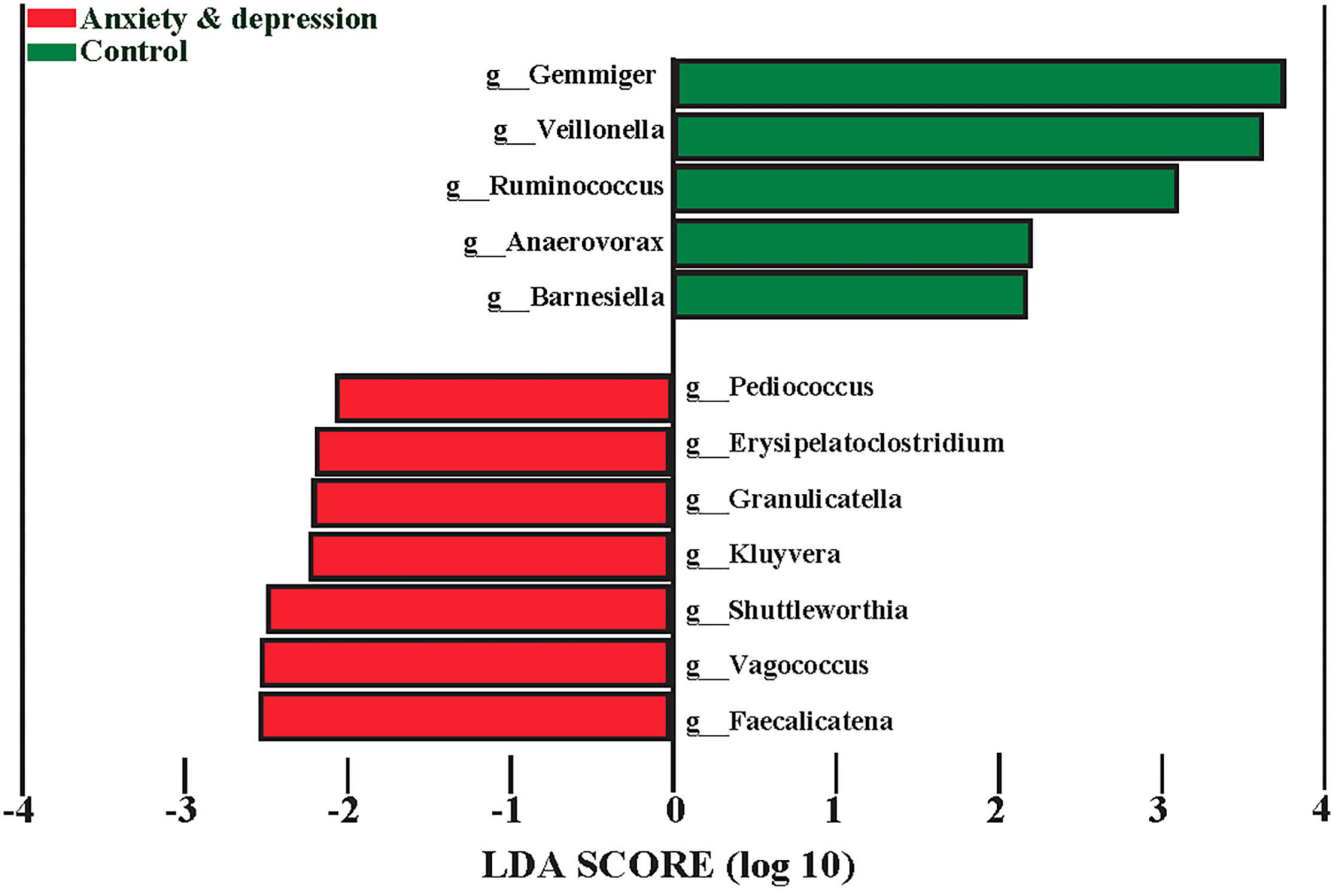
The microbiota associated with anxiety and depression from LEfSe.

The microbiota associated with anxiety and depression symptoms from LEfSe is shown in Figure 4. Screeners with positive anxiety and depression symptoms had greater abundances of *Pediococcus, Erysipelatoclostridium, Granulicatella, Kluyvera, Shuttleworthia, Vagococcus, Faecalicatena*, and lower greater abundances of *Gemmiger, Veillonella, Ruminococcus, Anaerovorax*, and *Barnesiella* at the genus level. Compared with controls, screeners with anxiety and depression symptoms had a less relative abundance of *Gemmiger* (1.4 vs. 2.3%, *P* = 0.025), *Ruminococcus* (0.6 vs. 0.8%, *P* = 0.037), and *Veillonella* (0.6 vs. 1.3%, *P* = 0.020) at genus level (see Supplementary Figure 3 for more details).

## Discussion

In this study, we investigated the microbial characterization of participants in gastrointestinal cancer screening and identified psychological distress-associated gut microbiota. The microbial environments of screeners all comprised primarily of *Firmicutes, Bacteroidetes*, and *Proteobacteria* at the phylum level and *Faecalibacterium, Roseburia*, and *Prevotella* at the genus level. Compared with the controls, the microbial characterization of screeners was distinct among participants with anxiety and depression, and the ranking of the top five genera in the anxiety and depression group changed. There was a lower relative abundance of *Gemmiger, Ruminococcus*, and *Veillonella* in participants with anxiety and depression, which was also reflected by the decreased alpha diversity in screeners who suffered psychological distress, although the difference was not significant. The findings filled the gap in the field of screening and contribute to a better understanding of endoscopic screening for gastrointestinal cancer and psychological distress, which would provide innovative strategies for relieving anxiety and depression and the optimization and implementation of endoscopic screening programs for upper gastrointestinal cancer in China.

It was evidently clear from the findings that participation in endoscopic screening may increase screeners' anxiety and depression symptoms. Current national cancer screening recommendations referenced the potential mental harm owing to cancer screening (11). The role of psychological status on screening has been seriously underestimated (12, 13). The psychological impact of the screening procedure itself is a common problem in all cancer screenings, but the psychological problem caused by endoscopic screening is more prominent due to its invasive nature, which presents a challenge to screeners' psychological states and emotions, increasing anxiety and depression levels. Taking esophageal cancer as an example, on the one hand, waiting for an invasive endoscopic examination may trigger or increase anxiety and depression levels. On the other hand, screeners are worried about screening results. Even low-risk grade health states (e.g., mild dysplasia and moderate dysplasia) are screened and diagnosed, and the risk of esophageal cancer is nearly 3–10 times higher than that of normal people. In this situation, patients may be scared of malignant deterioration and metastasis. Low-risk grade health states, such as moderate dysplasia, had ~28 times higher esophageal cancer incidence than normal individuals (25). In this case, it is difficult for patients to accept the fact in a short time, which may act as a serious stressor and stimulation of life-stress events, especially for patients who have been screened for EC and precancerous lesions. Considering cancer progression, recurrence, and prognosis, they are prone to distress (26).

Although debate is ongoing, the microbiota-gut-brain axis is becoming as significant as the microbiota for monitoring bidirectional gut-brain communication pathways (27). Reliable evidence has demonstrated the relationship among brain cognitive function, mood, and intestinal flora (4, 5). Reviews have shown that germ-free animals and animals with pathogenic bacterial infections played a vital role in the intestinal microbiota in the modulation of mood and cognition (27). Growing evidence indicates that the gastrointestinal microbiota is connected with anxiety and depression disorders. A wide range of studies consistently proposed that anxiety and depression impaired microbial characterization and diversity (8, 9). The study found that subjects with anxiety and depression had lower alpha diversity, although no significant differences were observed. The results were consistent with findings from previous research that the diversity and abundance of intestinal flora in patients with anxiety and depression decreased overall (9, 28). An important systematic review showed that α and β diversity were inconsistent. It indicated that the difference of bacterial taxa related to disorders may be manifested in a higher abundance of prion-flammatory species (e.g., *Enterobacteriaceae*) and lower bacteria producing short-chain fatty acid (e.g., *Faecalibacterium*) (10). Strong and consistent evidence has shown that *Firmicutes* and *Bacteroidetes* are dominant in human intestinal microbial flora (29). The change was inconspicuous in our study due to the relatively small sample size.

Several studies found consistent taxonomic differences among participants with generalizing anxiety disorders or depression relative to healthy controls, including higher *Bacteroidetes* and *Proteobacteria* and lower *Firmicutes* at the phylum level (10, 28, 30) and higher *Prevotella* and lower *Faecalibacterium* at the genus level (31), which is consistent with our results. Although these studies have found that the fecal flora of depressed patients is different from that of healthy individuals, the specific difference may vary, which may be related to the diagnostic criteria of research, inclusion criteria, and fecal detection methods (32). Animal experiments also found that the flora composition of depressed animals was similar to that of depressed patients, such as increased *Bacteroidetes* and decreased *Firmicutes* (33). However, several studies found the *Lactobacillus* and *Bifidobacterium* were decreased in depressed patients or animal models, which was not found in our study. It may be affected by the selection of subjects, inclusion criteria, and sample size.

In addition, we found that the specific genera *Gemmiger, Ruminococcus*, and *Veillonella* decreased in participants with anxiety and depression. Song et al. (34) proposed that a higher abundance of *Bacteroides* was linked with a higher fear of cancer recurrence. The relative abundance of *Gemmiger* in the anxiety and depression group was similar to that in another study related to diarrhea-predominant irritable bowel syndrome (2.4%) (35). In addition, Aranaz's study showed that the abundance of *Gemmiger* decreased in subjects with a higher inflammatory index (36). Similar results were found in a systematic review in which a reduced abundance of *Ruminococcus* was observed in depressed people (37). Evidence has shown that the high abundance of *Parabacteroide, Oscillibacter, Paraprevotella, Veillonella, Klebsiella*, and *Desulfovibrio* in patients with depression may demonstrate the role of flora in the emergence of depression (38). These results indicated that *Gemmiger, Ruminococcus*, and *Veillonella* may be the characteristic and specific genus of high-risk groups and vulnerable participants of anxiety and depression.

A growing body of evidence intriguingly suggests that the microbiota composition of individuals may affect their susceptibility to anxiety and depression (27). A key study presented the role of mouse microbiota transplantation in detecting the microbiota-gut-brain axis (39). A landmark study showed that sterile mice changed the function of the hypothalamic–pituitary–adrenal axis (HPA), which can be reversed by inhabiting specific bacterial strains early in life (40). The mechanism of how microbiota influences gut-brain signaling may be associated with the pathophysiology of anxiety and depression by delivering peripheral inflammation to the central nerve (28). These mechanisms may include modulating microbial composition, activating immunity, transducing vagal signals, alternating tryptophan metabolism, and producing specific microbial neuroactive metabolites (30).

In fact, due to the lack of longitudinal investigation in this study, we do not know how long psychological distress-associated gut microbiota would persist, and longitudinal investigations are sparse. First, we measured the symptoms of anxiety and depression with the GAD-7 and PHQ-9 in the past 2 weeks. Second, the intestinal flora was greatly influenced by diet, lifestyle, geography, and age, and the composition was dynamic and fluctuating (41, 42). Large-scale population studies found that antibiotics used in anti-infective treatment significantly increased the risk of individual psychological diseases such as anxiety and depression (43, 44). Once the microbiota becomes unbalanced, alteration may occur to the microenvironment and then lead to gastrointestinal diseases and even cancer (6, 7). Experimental evidence has shown that the human gut flora affects the occurrence and progression of gastrointestinal tumors by activating carcinogenic signaling pathways, producing tumor-promoting metabolites and inhibiting antitumor immune responses (45). In addition, evidence from saliva and tissues showed that oral flora may act as potential risk factors for oral and gastrointestinal cancer (7, 46), but there were differences in flora changes among various studies. *Fusobacterium nucleatum* mainly inhabits the oral cavity and causes periodontal disease, which may promote the aggressive behaviors of tumors by activating chemokines (e.g., CCL20) in esophageal cancer tissues (47). Therefore, given the important role of the gut microbiome in maintaining homeostasis, a better understanding of the microbiome in cancer screeners is increasingly important. We can develop innovative cancer prevention and therapeutic strategies by targeting the gut microbiota. The human intestinal microbiome plays an important role in cancer screening, especially for gastrointestinal cancer (48). Meta-analysis indicated that fecal bacteria and oral flora act as promising biomarkers for the noninvasive diagnosis of gastrointestinal cancer (49).

Since the gastrointestinal microbiota is altered through the rational use of prebiotics, probiotics, and antibiotics (50), the relationship between mental disorders, the microbiota, and tumors may be of clinical significance and implications. The novel concept of the microbiota-gut-brain axis indicated that regulation of the intestinal flora may be a feasible strategy to develop innovative therapeutics for psychological distress. These mechanisms may clear the way for microbial-based psychotherapies. Mind-altering microorganisms refer to the gut microbiota that could alter the brain and behavior. An important study showed that the potential probiotic could regulate behaviors related to anxiety and depressive disorders and alter central levels of γ-aminobutyric acid receptors (51). Compared with the placebo control group, probiotic Bifidobacterium longum and Lactobacillus helveticus reduced depression scores and altered the brain activity of patients with anxiety and depression (52). An important study consistently confirmed that probiotics help prevent and relieve depression disorders (31). In addition, clinical studies found that taking prebiotics daily for 3 weeks reduced the activation of the cortex caused by negative information, thereby reducing anxiety-like and depressive-like behaviors (53).

The pattern of intestinal microbiota changed significantly in patients with anxiety and depression. Further evidence is needed to translate microbiome findings into innovative clinical treatments to improve therapeutic effects in patients with mental disorders. Anxiety and depression have the characteristics of a low treatment rate, poor compliance, and recurrence, and this study provides a new promising research direction to improve psychological distress, namely mind-altering microorganisms. This suggests that we can attempt to explore probiotics, prebiotics, and other microecological agents to regulate the balance and homeostasis of intestinal flora in cancer screening progress, which contributes to optimizing screening and maximizing the net benefits of cancer screening. However, little clinical and large-scale research on probiotics and prebiotics has been used in treatment strategies. To date, the selection of probiotics is relatively random and difficult to predict, and it is hard to show stable efficacy in different groups. There are some shortcomings in the existing studies, such as a small sample size and poor contrast in the use of probiotics. Therefore, more randomized controlled trials are needed to further verify the efficacy of probiotics and prebiotics.

Adequate evidence has shown the bidirectional relationship between cancer and intestinal microbiota (54), such as colorectal cancer and malignant gastrointestinal diseases. Evidence highlighted the multifaceted role of the intestinal microbiota in cancer. The occurrence of cancer is usually accompanied by inflammation and leads to microbial alteration and disorder of the intestinal microbial environment, such as increased abundance of *Escherichia coli* and *Fusobacterium nucleatum* in colorectal cancer (54, 55). Conversely, the unbalanced microbiota could also cause a proinflammatory microenvironment and DNA damage, increasing the risk of cancer and deteriorating the prognosis, such as *Helicobacter pylori*, and invasive *Escherichia coli*. Further studies are needed to decipher whether there is a synergistic effect of microbial and psychological distress in tumor promotion, which may be exploited therapeutically in the future.

To our knowledge, first, this is the first study to explore the microbial characterization of participants in gastrointestinal cancer screening, filling the gap in the field of screening. This perspective is innovative and provides new ideas and optimization strategies for the comprehensive evaluation of cancer screening. Second, the included subjects were from the same regions, with similar dietary patterns, and the controls were matched by age and sex, which makes the findings more objective and credible to some extent. Some limitations of our work should be acknowledged. First, the stratified analysis of anxiety and depression separately was confined by the relatively small sample size. This may lead to no significant difference on some microbial characterizations and diversity, but the trend of our results provided scientific references in this field. Our exploratory study largely provides clues from a novel perspective. This study is an attempt to explore the microbial characterizations of screeners. Larger studies will be needed to reduce the uncertainty and confirm our associations. Second, we did not have specimens from non-screeners as bank controls. Third, selection bias may exist, and the sample may not be representative of all screeners. Further multicenter, large-scale, and prospective cohort studies, randomized controlled trials, and clinical trials are needed to validate the results. Fourth, anxiety and depression symptoms were evaluated in the study, not a clinical diagnosis of anxiety and depression disorders. In the future, psychiatrists could be considered in the screening process. Finally, considering the goal of this study and other factors (e.g., economy, efficiency, and data processing), we chose 16S rRNA gene sequencing. In the future, full metagenomics could be applied for further exploration of mechanisms, pathway in-depth, and functional prediction analysis.

## Conclusions

Differing microbial characterization among participants with anxiety and depression was found in the endoscopic screening of upper gastrointestinal cancer in China. *Gemmiger, Ruminococcus*, and *Veillonella* were informative for psychological distress in cancer screening and have potential clinical implications for mental disorders, which provides references for optimizing cancer screening and minimizing psychological harm. The results should be explained cautiously, and more large-scale, prospective cohort studies are needed in the future to validate the results and further explore biological mechanisms and the relationship among gut microbiota, psychological distress, and cancer risk in cancer screening.

## Data Availability Statement

The data presented in the study are deposited in the the Genome Sequence Archive (GSA) repository, accession number CRA005126.

## Ethics Statement

This study was approved by the Institutional Review Board of the Cancer Hospital of the Chinese Academy of Medical Sciences (No. 21/030-2701; 16-171/1250). The patients/participants provided their written informed consent to participate in this study.

## Author Contributions

This research was designed by WW. JZ drafted the manuscript. ML, DS, and JZ collected the related data and materials. JZ analyzed and interpreted the data. JZ, SM, and WW revised the manuscript. All authors contributed to the article and approved the submitted version.

## Funding

This work was supported by the National Natural Science Foundation of China (Grant/Award Number: 81974493) and the National Key Research and Development Program (Precision Medicine Research) (Grant/Award Number: 2016YFC0901400, 2016YFC0901404).

## Conflict of Interest

The authors declare that the research was conducted in the absence of any commercial or financial relationships that could be construed as a potential conflict of interest.

## Publisher's Note

All claims expressed in this article are solely those of the authors and do not necessarily represent those of their affiliated organizations, or those of the publisher, the editors and the reviewers. Any product that may be evaluated in this article, or claim that may be made by its manufacturer, is not guaranteed or endorsed by the publisher.

## References

[1] Fung TC Olson CA Hsiao EY. Interactions between the microbiota, immune and nervous systems in health and disease. Nat Neurosci. (2017) 20:145–55. 10.1038/nn.4476 28092661 PMC6960010

[2] Clemente JC Ursell LK Parfrey LW Knight R. The impact of the gut microbiota on human health: an integrative view. Cell. (2012) 148:1258–70. 10.1016/j.cell.2012.01.035 22424233 PMC5050011

[3] Hooper LV Littman DR Macpherson AJ. Interactions between the microbiota and the immune system. Science. (2012) 336:1268–73. 10.1126/science.1223490 22674334 PMC4420145

[4] Muller PA Schneeberger M Matheis F Wang P Kerner Z Ilanges A. Microbiota modulate sympathetic neurons *via* a gut-brain circuit. Nature. (2020) 583:441–6. 10.1038/s41586-020-2474-7 32641826 PMC7367767

[5] Sherwin E Bordenstein SR Quinn JL Dinan TG Cryan JF. Cryan. Microbiota and the social brain. Science. (2019) 366:eaar2016. 10.1126/science.aar2016 31672864

[6] Garrett WS. Cancer and the microbiota. Science. (2015) 348:80–6. 10.1126/science.aaa4972 25838377 PMC5535753

[7] Shao D Vogtmann E Liu A Qin J Chen W Abnet CC. Microbial characterization of esophageal squamous cell carcinoma and gastric cardia adenocarcinoma from a high-risk region of China. Cancer. (2019) 125:3993–4002. 10.1002/cncr.32403 31355925 PMC7285383

[8] Foster JA McVey Neufeld KA. Gut-brain axis: how the microbiome influences anxiety and depression. Trends Neurosci. (2013) 36:305–12. 10.1016/j.tins.2013.01.005 23384445

[9] Jiang H Ling Z Zhang Y Mao H Ma Z Yin Y. Altered fecal microbiota composition in patients with major depressive disorder. Brain Behav Immun. (2015) 48:186–94. 10.1016/j.bbi.2015.03.016 25882912

[10] Simpson CA Diaz-Arteche C Eliby D Schwartz OS Simmons JG Cowan CSM. The gut microbiota in anxiety and depression: a systematic review. Clin Psychol Rev. (2021) 83:101943. 10.1016/j.cpr.2020.101943 33271426

[11] Lauby-Secretan B Scoccianti C Loomis D Benbrahim-Tallaa L Bouvard V Bianchini F. Breast cancer screening viewpoint of the IARC Working Group. N Engl J Med. (2015) 372:2353–8. 10.1056/NEJMsr1504363 26039523

[12] Craanen ME Kuipers EJ. Advantages and disadvantages of population screening for cancer and surveillance of at-risk groups. Best Pract Res Clin Gastroenterol. (2001) 15:211–6. 10.1053/bega.2000.0170 11355912

[13] Nelson HD O'Meara ES Kerlikowske K Balch S Miglioretti D. Factors associated with rates of false-positive and false-negative results from digital mammography screening: an analysis of registry data. Ann Intern Med. (2016) 164:226–35. 10.7326/M15-0971 26756902 PMC5091936

[14] Walters WA Caporaso JG Lauber CL Berg-Lyons D Fierer N Knight R. PrimerProspector: *de novo* design and taxonomic analysis of barcoded polymerase chain reaction primers. Bioinformatics. (2011) 27:1159–61. 10.1093/bioinformatics/btr087 21349862 PMC3072552

[15] Caporaso JG Lauber CL Walters WA Berg-Lyons D Huntley J Fierer N. Ultrahigh-throughput microbial community analysis on the Illumina HiSeq and MiSeq platforms. ISME J. (2012) 6:1621–4. 10.1038/ismej.2012.8 22402401 PMC3400413

[16] Bolyen E Rideout JR Dillon MR Bokulich NA Abnet CC Al-Ghalith GA. Reproducible, interactive, scalable and extensible microbiome data science using QIIME 2. Nat Biotechnol. (2019) 37:852–7. 10.1038/s41587-019-0209-9 31341288 PMC7015180

[17] Callahan BJ McMurdie PJ Rosen MJ Han AW Johnson AJ Holmes SP. DADA2: High-resolution sample inference from Illumina amplicon data. Nat Methods. (2016) 13:581–3. 10.1038/nmeth.3869 27214047 PMC4927377

[18] DeSantis TZ Hugenholtz P Larsen N Rojas M Brodie EL Keller K. Greengenes, a chimera-checked 16S rRNA gene database and workbench compatible with ARB. Appl Environ Microbiol. (2006) 72:5069–72. 10.1128/AEM.03006-05 16820507 PMC1489311

[19] Kroenke K Spitzer RL Williams JB Monahan PO Löwe B. Anxiety symptoms in primary care: prevalence, impairment, comorbidity, and detection. Ann Intern Med. (2007) 146:317–25. 10.7326/0003-4819-146-5-200703060-00004 17339617

[20] Tong X An D McGonigal A Park SP Zhou D. Validation of the Generalized Anxiety Disorder-7 (GAD-7) among Chinese people with epilepsy. Epilepsy Res. (2016) 120:31–6. 10.1016/j.eplepsyres.2015.11.019 26709880

[21] Kroenke K Spitzer RL Williams JB. The PHQ-9: validity of a brief depression severity measure. J Gen Intern Med. (2001) 16:606–13. 10.1046/j.1525-1497.2001.016009606.x 11556941 PMC1495268

[22] Levis B Benedetti A Thombs BD. Accuracy of Patient Health Questionnaire-9 (PHQ-9) for screening to detect major depression: individual participant data meta-analysis. Br Med J. (2019) 365:l1476. 10.1136/bmj.l1476 30967483 PMC6454318

[23] Löwe B Kroenke K Herzog W Gräfe K. Measuring depression outcome with a brief self-report instrument: sensitivity to change of the Patient Health Questionnaire (PHQ-9). J Affect Disord. (2004) 81:61–6. 10.1016/S0165-0327(03)00198-8 15183601

[24] Segata N Izard J Waldron L Gevers D Miropolsky L Garrett WS. Metagenomic biomarker discovery and explanation. Genome Biol. (2011) 12:R60. 10.1186/gb-2011-12-6-r60 21702898 PMC3218848

[25] Wei WQ Hao CQ Guan CT Song GH Wang M Zhao DL. Esophageal histological precursor lesions and subsequent 8.5-year cancer risk in a population-based prospective study in China. Am J Gastroenterol. (2020) 115:1036–44. 10.14309/ajg.0000000000000640 32618654 PMC7477846

[26] Zhu J Zhou Y Ma S Chen R Xie S Liu Z. The association between anxiety and esophageal cancer: a nationwide population-based study. Psychooncology. (2021) 30:321–30. 10.1002/pon.5580 33098157

[27] Cryan JF Dinan TG. Mind-altering microorganisms: the impact of the gut microbiota on brain and behaviour. Nat Rev Neurosci. (2012) 13:701–12. 10.1038/nrn3346 22968153

[28] Jiang HY Zhang X Yu ZH Zhang Z Deng M Zhao JH. Altered gut microbiota profile in patients with generalised anxiety disorder. J Psychiatr Res. (2018) 104:130–6. 10.1016/j.jpsychires.2018.07.007 30029052

[29] Eckburg PB Bik EM Bernstein CN Purdom E Dethlefsen L Sargent M. Diversity of the human intestinal microbial flora. Science. (2005) 308:1635–8. 10.1126/science.1110591 15831718 PMC1395357

[30] Chen YH Bai J Wu D Yu SF Qiang XL Bai H. Association between fecal microbiota and generalised anxiety disorder: severity and early treatment response. J Affect Disord. (2019) 259:56–66. 10.1016/j.jad.2019.08.014 31437702

[31] Valles-Colomer M Falony G Darzi Y Tigchelaar EF Wang J Tito RY. The neuroactive potential of the human gut microbiota in quality of life and depression. Nat Microbiol. (2019) 4:623–32. 10.1038/s41564-018-0337-x 30718848

[32] Zheng P Zeng B Zhou C Liu M Fang Z Xu X. Gut microbiome remodeling induces depressive-like behaviors through a pathway mediated by the host's metabolism. Mol Psychiat. (2016) 21:786–96. 10.1038/mp.2016.44 27067014

[33] Kaser M Zaman R Sahakian BJ. Cognition as a treatment target in depression. Psychol Med. (2017) 47:987–9. 10.1017/S0033291716003123 27938430

[34] Song BC Bai J. Microbiome-gut-brain axis in cancer treatment-related psychoneurological toxicities and symptoms: a systematic review. Support Care Cancer. (2021) 29:605–17. 10.1007/s00520-020-05739-9 32918608 PMC7769970

[35] Jia Q Zhang L Zhang J Pei F Zhu S Sun Q. Fecal microbiota of diarrhea-predominant irritable bowel syndrome patients causes hepatic inflammation of germ-free rats and berberine reverses it partially. Biomed Res Int. (2019) 2019:4530203. 10.1155/2019/4530203 31073525 PMC6470425

[36] Aranaz P Ramos-Lopez O Cuevas-Sierra A Martinez JA Milagro FI Riezu-Boj JI. A predictive regression model of the obesity-related inflammatory status based on gut microbiota composition. Int J Obes (Lond). (2021) 45:2261–8. 10.1038/s41366-021-00904-4 34267323

[37] Cheung SG Goldenthal AR Uhlemann AC Mann JJ Miller JM Sublette ME. Systematic review of gut microbiota and major depression. Front Psychiatry. (2019) 10:34. 10.3389/fpsyt.2019.00034 30804820 PMC6378305

[38] Barandouzi ZA Starkweather AR Henderson WA Gyamfi A Cong XS. Altered composition of gut microbiota in depression: a systematic review. Front Psychiatry. (2020) 11:541. 10.3389/fpsyt.2020.00541 32587537 PMC7299157

[39] Bercik P Denou E Collins J Jackson W Lu J Jury J. The intestinal microbiota affect central levels of brain-derived neurotropic factor and behavior in mice. Gastroenterology. (2011) 141:599–609.e3. 10.1053/j.gastro.2011.04.052 21683077

[40] Sudo N Chida Y Aiba Y Sonoda J Oyama N Yu XN. Postnatal microbial colonization programs the hypothalamic-pituitary-adrenal system for stress response in mice. J Physiol. (2004) 558:263–75. 10.1113/jphysiol.2004.063388 15133062 PMC1664925

[41] Strasser B Wolters M Weyh C Krüger K Ticinesi A. The effects of lifestyle and diet on gut microbiota composition, inflammation and muscle performance in our aging society. Nutrients. (2021) 13:2045. 10.3390/nu13062045 34203776 PMC8232643

[42] Yatsunenko T Rey FE Manary MJ Trehan I Dominguez-Bello MG Contreras M. Human gut microbiome viewed across age and geography. Nature. (2012) 486:222–27. 10.1038/nature11053 22699611 PMC3376388

[43] Lurie I Yang YX Haynes K Mamtani R Boursi B. Antibiotic exposure and the risk for depression, anxiety, or psychosis: a nested case-control study. J Clin Psychiatry. (2015) 76:1522–8. 10.4088/JCP.15m09961 26580313

[44] Köhler O Petersen L Mors O Mortensen PB Yolken RH Gasse C. Infections and exposure to anti-infective agents and the risk of severe mental disorders: a nationwide study. Acta Psychiat Scand. (2017) 135:97–105. 10.1111/acps.12671 27870529

[45] Zitvogel L Ayyoub M Routy B Kroemer G. Microbiome and anticancer immunosurveillance. Cell. (2016) 165:276–87. 10.1016/j.cell.2016.03.001 27058662

[46] Chen Y Chen X Yu H Zhou H Xu S. Oral microbiota as promising diagnostic biomarkers for gastrointestinal cancer: a systematic review. Onco Targets Ther. (2019) 12:11131–44. 10.2147/OTT.S230262 31908481 PMC6927258

[47] Baba Y Iwatsuki M Yoshida N Watanabe M Baba H. Review of the gut microbiome and esophageal cancer: pathogenesis and potential clinical implications. Ann Gastroenterol Surg. (2017) 1:99–104. 10.1002/ags3.12014 29863142 PMC5881342

[48] Kashyap S Pal S Chandan G Saini V Chakrabarti S Saini NK. Understanding the cross-talk between human microbiota and gastrointestinal cancer for developing potential diagnostic and prognostic biomarkers. Semin Cancer Biol. (2021) 8:S1044-579X(21)00121–8. 10.1016/j.semcancer.2021.04.020 33971261

[49] Wirbel J Pyl PT Kartal E Zych K Kashani A Milanese A. Meta-analysis of fecal metagenomes reveals global microbial signatures that are specific for colorectal cancer. Nat Med. (2019) 25:679–89. 10.1038/s41591-019-0406-6 30936547 PMC7984229

[50] Ianiro G Tilg H Gasbarrini A. Antibiotics as deep modulators of gut microbiota: between good and evil. Gut. (2016) 65:1906–5. 10.1136/gutjnl-2016-312297 27531828

[51] Bravo JA Forsythe P Chew MV Escaravage E Savignac HM Dinan TG. Ingestion of Lactobacillus strain regulates emotional behavior and central GABA receptor expression in a mouse *via* the vagus nerve. Proc Natl Acad Sci U S A. (2011) 108:16050–5. 10.1073/pnas.1102999108 21876150 PMC3179073

[52] Pinto-Sanchez MI Hall GB Ghajar K Nardelli A Bolino C Lau JT. Probiotic *Bifidobacterium longum* NCC3001 reduces depression scores and alters brain activity: a pilot study in patients with irritable bowel syndrome. Gastroenterology. (2017) 153:448–59.e8. 10.1053/j.gastro.2017.05.003 28483500

[53] Schmidt K Cowen PJ Harmer CJ Tzortzis G Errington S Burnet PW. Prebiotic intake reduces the waking cortisol response and alters emotional bias in healthy volunteers. Psychopharmacology (Berl). (2015) 232:1793–801. 10.1007/s00213-014-3810-0 25449699 PMC4410136

[54] Tilg H Adolph TE Gerner RR Moschen AR. The intestinal microbiota in colorectal cancer. Cancer Cell. (2018) 33:954–64. 10.1016/j.ccell.2018.03.004 29657127

[55] Irrazábal T Belcheva A Girardin SE Martin A Philpott DJ. The multifaceted role of the intestinal microbiota in colon cancer. Mol Cell. (2014) 54:309–20. 10.1016/j.molcel.2014.03.039 24766895

