# Supplementary material for: Altered Fecal Microbiota Signatures in Patients With Anxiety and Depression in the Gastrointestinal Cancer Screening: A Case-Control Study
Source: Front Psychiatry. 2021 Nov 8;12:757139. doi: 10.3389/fpsyt.2021.757139 (PMC8607523; doi:10.3389/fpsyt.2021.757139)
Supplement: Supplementary file 1 [file Table_1.DOCX]

**Supplementary Table 1 |** Alpha diversity and composition of microbial taxa for anxiety & depression group and control group

|  |  | **All (rank)** | **Anxiety & depression group (rank)** |  | **Control  group (rank)** | *P* |
| --- | --- | --- | --- | --- | --- | --- |
| **Alpha diversity** | |  |  |  |  |  |
|  | Evenness index | 0.67 | 0.68 |  | 0.66 | 0.508 |
|  | Observed OTUs | 135.77 | 122.35 |  | 143.24 | 0.065 |
|  | Shannon index | 4.69 | 4.66 |  | 4.70 | 0.849 |
|  | Chao1 index | 142.43 | 127.35 |  | 149.98 | 0.055 |
| **The top 5 phyla, %** | |  |  |  |  |  |
|  | *Firmicutes* | 71.2 (1) | 66.6 (1) |  | 73.4 (1) | 0.240 |
|  | *Bacterroidetes* | 14.6 (2) | 15.5 (2) |  | 14.1 (2) | 0.620 |
|  | *Proteobacteria* | 5.8 (3) | 7.8 (3) |  | 4.7 (3) | 0.100 |
|  | *Actinobacteria* | 2.8 (4) | 2.2 (4) |  | 3.1 (4) | 0.541 |
|  | *Unknown* | 1.8 (5) | 1.6 (5) |  | 2.0 (5) | 0.809 |
| **The top 5 genera, %** | |  |  |  |  |  |
|  | *Faecalibacterium* | 11.3 (1) | 10.5 (2) |  | 11.8 (1) | 0.470 |
|  | *Roseburia* | 10.4 (2) | 9.2 (4) |  | 10.9 (2) | 0.600 |
|  | *Prevotella* | 10.3 (3) | 10.9 (1) |  | 10.0 (3) | 0.600 |
|  | *Blautia* | 10.0 (4) | 10.2 (3) |  | 9.8 (4) | 0.720 |
|  | *Escherichia* | 3.0 (5) | 3.9 (5) |  | 2.6 (5) | 0.679 |
