# Supplementary figures and images for: Altered Fecal Microbiota Signatures in Patients With Anxiety and Depression in the Gastrointestinal Cancer Screening: A Case-Control Study
Source: Front Psychiatry. 2021 Nov 8;12:757139. doi: 10.3389/fpsyt.2021.757139 (PMC8607523; doi:10.3389/fpsyt.2021.757139)

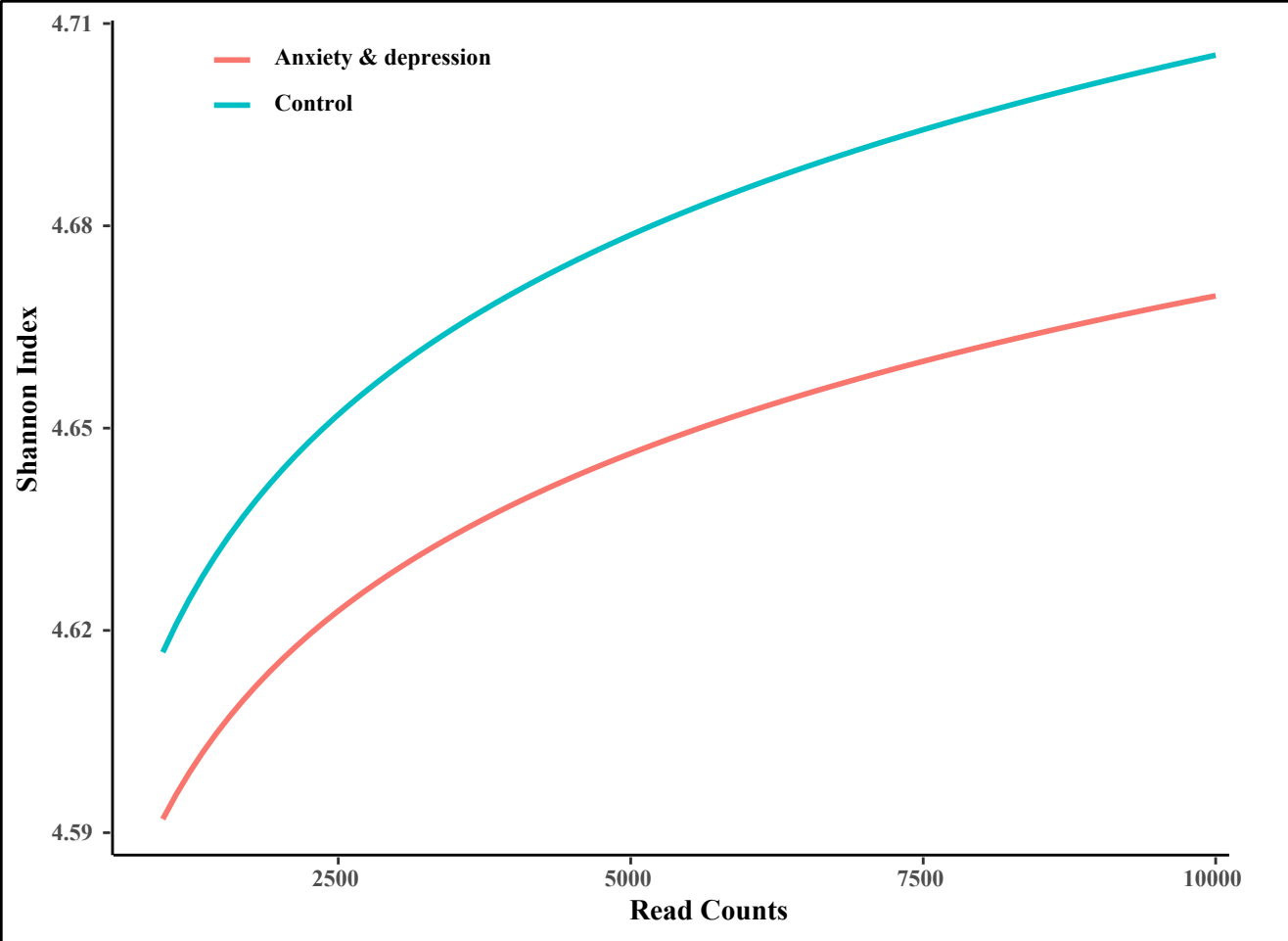

**Supplementary Fig. 1 The Shannon index rarefaction curve**

Supplement: Supplementary file 2 [file Data_Sheet_1.PDF]
