# Supplementary material for: Altered Fecal Microbiota Signatures in Patients With Anxiety and Depression in the Gastrointestinal Cancer Screening: A Case-Control Study
Source: Front Psychiatry. 2021 Nov 8;12:757139. doi: 10.3389/fpsyt.2021.757139 (PMC8607523; doi:10.3389/fpsyt.2021.757139)
Supplement: Supplementary file 4 [file Data_Sheet_3.PDF]

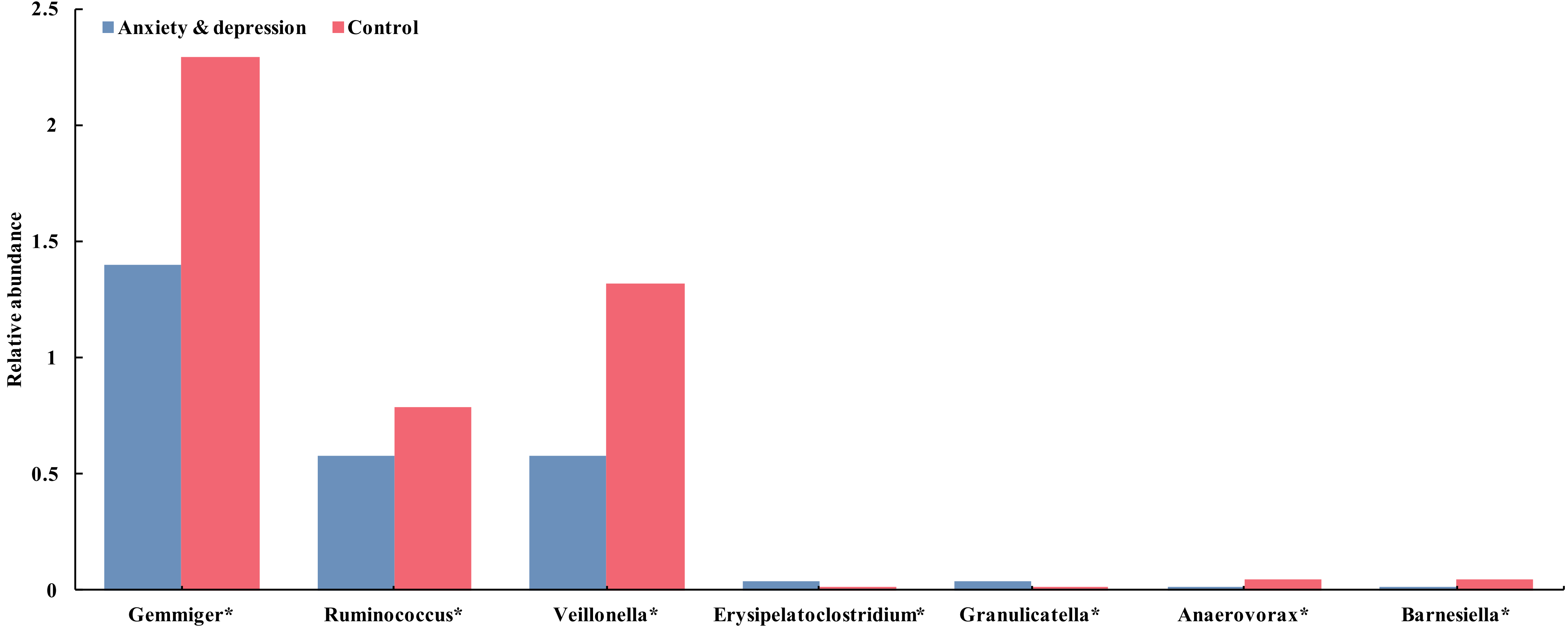

Supplementary Fig. 3 Comparison of relative abundance of characteristic genus between anxiety & depression group and control group
